# Thalamic quantitative susceptibility mapping and susceptibility source separation: a critical narrative review of evidence and interpretation

**DOI:** 10.3389/fneur.2026.1878540

**Published:** 2026-09-24

**Authors:** Sadegh Ghaderi, Maria Agnese Pirozzi, Minoo Sharbafshaaer, Francesca Trojsi, Sana Mohammadi, Seyed-Mohammad Fereshtehnejad

**Affiliations:** 1 Department of Neurology and Neurosurgery, Montreal Neurological Institute, McGill University, Montreal, QC, Canada; 2 McConnell Brain Imaging Centre, Montreal Neurological Institute, McGill University, Montreal, QC, Canada; 3 Ludmer Centre for Neuroinformatics and Mental Health, Montreal, QC, Canada; 4 Department of Advanced Medical and Surgical Sciences, MRI Research Center (DAMSS), University of Campania “Luigi Vanvitelli”, Naples, Italy; 5 Neurology Unit, First Division of Neurology and Neurophysiopathology, AOU University of Campania “Luigi Vanvitelli”, Naples, Italy; 6 Department of Medical Sciences, School of Medicine, Iran University of Medical Sciences, Tehran, Iran; 7 School of Medicine, Hamadan University of Medical Sciences, Hamadan, Iran; 8 Pacific Parkinson's Research Centre (PPRC), Djavad Mowafaghian Centre for Brain Health, University of British Columbia, Vancouver, BC, Canada; 9 Division of Neurology, Faculty of Medicine, University of British Columbia, Vancouver, BC, Canada; 10 Division of Clinical Geriatrics, Department of Neurobiology, Care Sciences and Society (NVS), Karolinska Institute, Stockholm, Sweden

**Keywords:** calcification, iron deposition, magnetic susceptibility, quantitative susceptibility mapping (QSM), thalamus, χ-separation

## Abstract

Quantitative susceptibility mapping (QSM) measures net tissue magnetic susceptibility, which can conceal coexisting contributions of opposite sign. This is relevant to the thalamus, where nuclei differ in cellular composition, myelinated fibers, and susceptibility contrast. This narrative review evaluates what QSM and magnetic susceptibility source separation, including χ-separation, currently establish about thalamic alterations in aging and neurological disease. The synthesis prioritizes human thalamic evidence and distinguishes it from methodological validation, findings outside the thalamus, and animal experiments. We use paramagnetic component susceptibility (PCS) and diamagnetic component susceptibility (DCS) as the principal notation, with PCS nonnegative and DCS nonpositive. These model-derived components describe magnetic behavior and do not independently identify iron, myelin, calcium, amyloid, or tau. Reduced thalamic bulk susceptibility is repeatedly reported in multiple sclerosis, whereas findings in Alzheimer's disease and Parkinson's disease are less consistent. Differences in acquisition, referencing, segmentation, cohort composition, and treatment of atrophy affect interpretation, although methodological variability does not explain every disagreement. Source separation can provide information concealed by net susceptibility, but depends on relaxation modeling, acquisition conditions, and reconstruction assumptions. Evidence for thalamic protein mapping, calcification of characterization, and treatment monitoring remains limited or indirect. PCS and DCS are research measurements with potential biological interpretability; standardized acquisition and processing, independent tissue validation, longitudinal replication, and clinically useful decision thresholds are required before they can be regarded as validated clinical biomarkers.

## 1 Introduction

The thalamus comprises anatomically and functionally distinct nuclei linked to sensory, motor, associative, and limbic networks (1–3). Its internal medullary lamina, intranuclear myelinated fibers, and interfaces with surrounding white matter create substantial variation in tissue composition within a small structure. A whole thalamus mean therefore combines compartments that may differ in baseline susceptibility and disease involvement. Thalamic susceptibility alterations have been reported in neurological disorders, but the interpretation of those alterations requires attention to both anatomy and measurement of assumptions (4, 5).

Conventional T2^*^-weighted imaging and susceptibility-weighted imaging (SWI) detect susceptibility-related signal changes, including hypointense hemorrhagic and calcified lesions, but magnitude contrast alone does not establish their composition (6, 7). QSM adds an estimate of net susceptibility relative to a specified reference. A positive or negative value can support magnetic differentiation under appropriate conditions, but neither sign is a chemical assay. In particular, the coexistence of magnetic contributions of opposite sign can yield a small net value despite appreciable contributions from both classes of source (8, 9).

The central question of this review is how much biological interpretation is justified by thalamic QSM and by model-derived susceptibility source separation. We distinguish sensitivity to a tissue change, greater interpretability through additional contrasts, and validation for a defined clinical decision. The ability to estimate two magnetic components does not automatically establish two pathological biomarkers. These distinctions are especially important when extrapolating from iron-rich basal ganglia, white-matter lesions, or experimental tissue to the mixed microstructure of thalamic nuclei.

Anatomical definition is a prerequisite for subnuclear interpretation (Figure 1). The histology-informed FreeSurfer atlas delineates 26 thalamic nuclei, whereas the original THOMAS framework parcellates 12 major nuclei using specialized structural contrast (3, 10). NextBrain provides a probabilistic histological atlas covering 333 brain regions, including thalamic subdivisions (1, 11). These label sets differ in granularity, boundaries, and supporting images; identically named regions should not be assumed to contain identical tissue. More labels do not necessarily confer greater measurement accuracy when nucleus dimensions approach the acquired voxel size. The subthalamic nucleus is anatomically distinct from the thalamus and is not a thalamic subnucleus.

**Figure 1:**
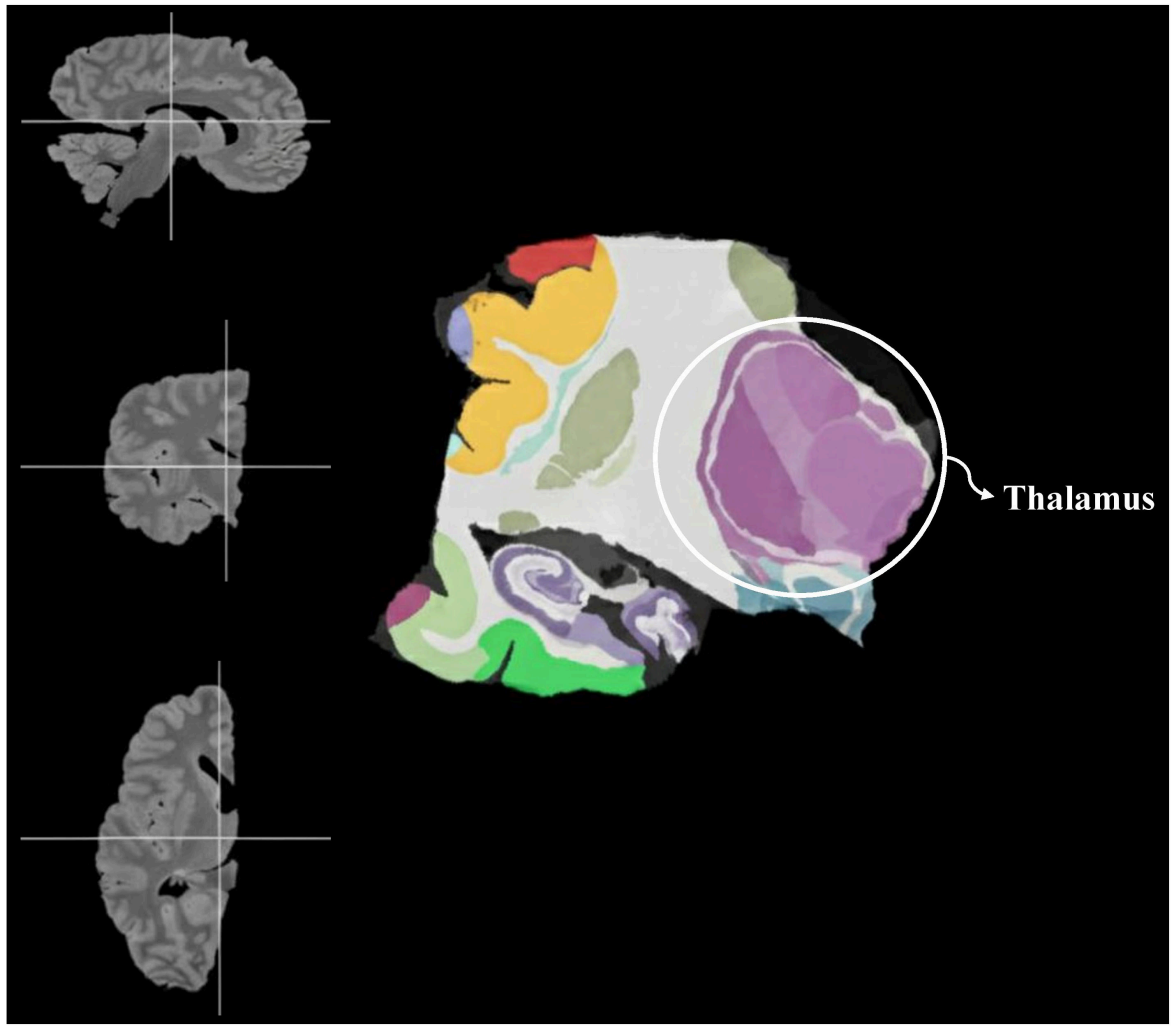
High-resolution segmentation of thalamic subnuclei. This figure illustrates the intricate architecture of the thalamus, which is not a monolithic structure but a dense collection of distinct nuclei (e.g., anterior, medial, and pulvinar groups). Accurate in vivo segmentation, facilitated by next-generation probabilistic atlases such as NextBrain, is essential for sub-regional QSM analysis. This high-granularity mapping allows for the assessment of selective vulnerability, where pathological processes like iron accumulation or calcification affect specific functional subdivisions differently (1, 3).

Registration from structural or GRE magnitude images to the susceptibility acquisition, followed by visual inspection of every thalamic boundary, is needed to assess label placement. Interpolation, registration error, disease-related deformation, and partial volume with the internal capsule or cerebrospinal fluid (CSF) may bias a small region disproportionately. A normative susceptibility atlas can assist localization but cannot replace subject-specific quality control or validate pathological composition (12).

Accordingly, we focus on thalamic measurement and interpretation in aging, Alzheimer's disease (AD), Parkinson's disease (PD), multiple sclerosis (MS), amyotrophic lateral sclerosis (ALS), and calcification. Deep learning, ultra-high-field MRI, and neurosurgical imaging are discussed only as far as they affect this objective. This is a narrative synthesis rather than a systematic review or a new meta-analysis.

### 1.1 Narrative review approach

Searches used web access to PubMed-indexed records, PubMed Central full texts, and primary publisher pages. Search concepts combined “thalamus” or “thalamic” with “quantitative susceptibility mapping”, “QSM”, “χ-separation”, “chi-separation”, or “susceptibility source separation”. Application-oriented searches added “multiple sclerosis”, “Alzheimer”, “Parkinson”, “amyotrophic lateral sclerosis”, “aging”, or “calcification”; targeted methodological checks used “atrophy”, “reference”, “segmentation”, and “static dephasing”. Searches by article title or DOI checked studies cited for key claims. The emphasis was on evidence relevant to interpreting thalamic measurements, rather than maximizing the number of included publications.

Eligible evidence comprised human MRI studies reporting thalamic or thalamic-subregional QSM or source-separated findings, relevant acquisition/reconstruction and atlas-validation studies, and experimental work needed to assess proposed tissue interpretations. Animal, *ex vivo*, and extra-thalamic studies were retained when they directly informed a mechanism or a stated limitation and were labeled as indirect evidence. Reviews supplied context, with primary reports prioritized for numerical and application-specific claims. Studies without a relevant thalamic or methodological contribution were not used to support clinical conclusions; conference or preprint observations were not treated as equivalent to established clinical evidence.

## 2 Measurement principles relevant to the thalamus

QSM reconstruction and source separation address different inverse problems. QSM estimates net susceptibility from the spatial field distribution. Source-separation methods introduce additional signal information and assumptions to estimate opposing contributions. Both steps can influence regional values, and a visually plausible map is insufficient evidence of quantitative accuracy (9, 13).

### 2.1 QSM acquisition, reconstruction, and referencing

The conventional QSM workflow comprises complex coil combination, phase unwrapping or field estimation, background-field removal, and dipole inversion (Figure 2) (13, 14). Sum-of-squares combination produces magnitude images and does not preserve the complex phase needed for QSM; phase-sensitive coil combination must account for coil-dependent phase offsets. Phase unwrapping resolves 2π discontinuities, using methods such as Laplacian unwrapping, SEGUE, or ROMEO (15–17). Echo timing, spatial resolution, motion, and signal-to-noise ratio influence these steps and should be reported alongside the reconstruction software and settings.

**Figure 2:**
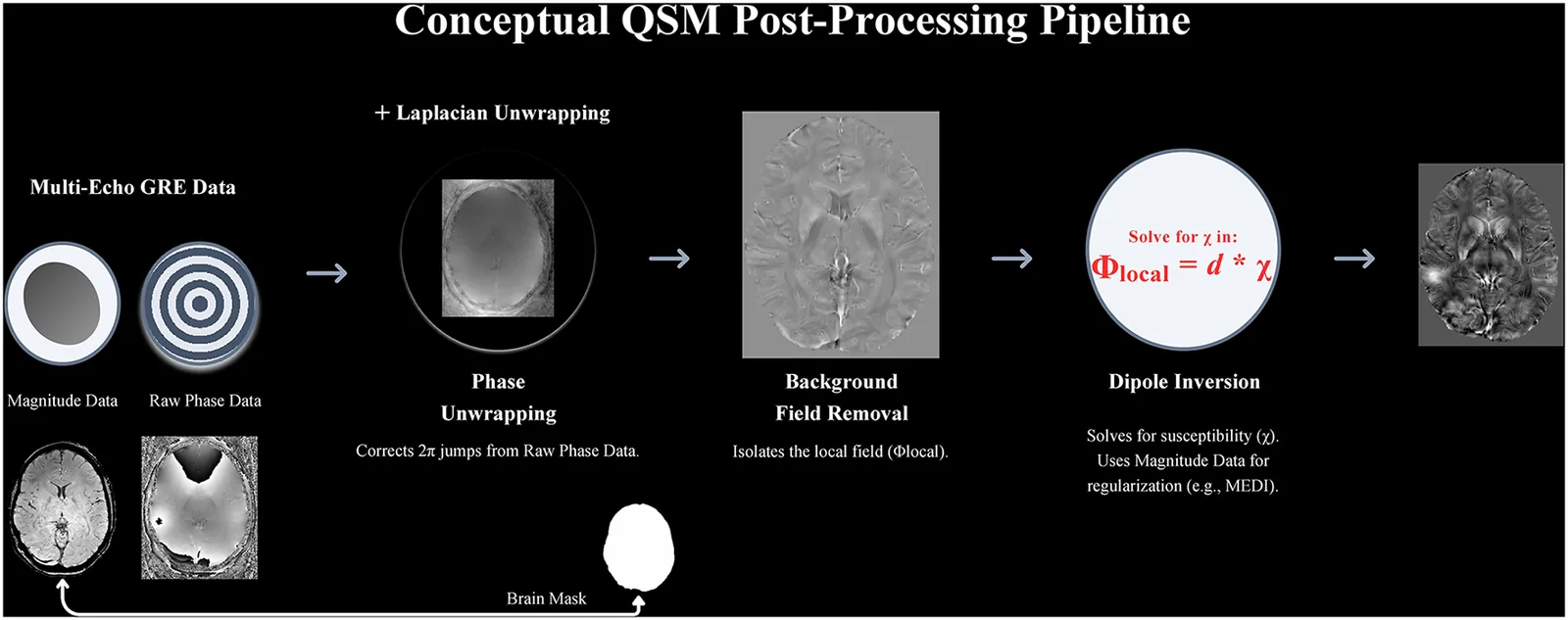
Conventional QSM processing pipeline. Multi-echo GRE acquisition supplies magnitude and phase data. Phase unwrapping corrects 2π discontinuities, background-field removal estimates the local tissue field, and dipole inversion estimates bulk susceptibility; magnitude information can support regularization, as in MEDI. In the schematic equation Φ local = d * χ, Φ local denotes the appropriately scaled local field (or phase normalized by gyromagnetic ratio, B0, and echo time), d is the dipole kernel, and * denotes spatial convolution. Raw phase is not numerically equal to susceptibility without this scaling. This figure describes QSM reconstruction, not susceptibility source separation.

Background-field removal reduces fields generated by sources outside the region of interest. Projection onto Dipole Fields uses an external-source projection; Laplacian Boundary Value methods impose harmonic boundary constraints, and SHARP-family methods exploit the spherical mean-value property of harmonic fields (18–21). They are not interchangeable implementations of one boundary-value problem. Mask erosion and regularization may remove tissue information as well as background fields. No algorithm is uniformly superior across all masks, susceptibility distributions, and acquisition conditions (13).

Dipole inversion is ill-conditioned because the Fourier-space dipole kernel approaches zero on a cone at approximately 54.7° to the main magnetic field. Truncated k-space division, morphology-enabled dipole inversion (MEDI), iterative least-squares methods, and STAR-QSM differ in their regularization and treatment of artifacts (22–27). Streak suppression may be accompanied by smoothing or attenuation of contrast. Consequently, apparent nucleus boundaries and regional effect sizes depend partly on the reconstruction and should not be interpreted as unfiltered tissue properties.

Deep-learning reconstruction can reduce computation and improve agreement with training targets (28–30). However, agreement with a reference to reconstruction does not establish chemical specificity or generalization to a different scanner, disease, or field strength. Multiple-orientation COSMOS improves conditioning of the dipole inversion, but remains an MRI reference method with its own acquisition and modeling limitations, rather than a histological ground truth (25, 31). For a thalamic review, the relevant question is whether a method preserves small-region quantitative differences in independent data, not simply whether its images appear sharper.

QSM values are generally expressed in parts per million (ppm) or parts per billion (ppb) relative to a stated reference, because the spatially uniform susceptibility offset is not determined by the measured field (13, 32–34). If the reference changes with aging or disease, a target-to-reference difference can change without a corresponding alteration confined to the target. White-matter references may be affected by demyelination and orientation; CSF estimates may be affected by partial volume and artifacts; a whole-brain reference may contain widespread pathology. Reporting no explicit reference does not make the result absolute. Reference choice and sensitivity analyses using a second plausible reference should be reported where reference stability is uncertain.

### 2.2 Opposing contributions and consistent notation

Throughout this review, PCS denotes paramagnetic component susceptibility (PCS ≥ 0), and DCS denotes diamagnetic component susceptibility (DCS ≤ 0); |DCS| denotes its nonnegative magnitude. For the additive model below, they are voxel-effective contributions expressed in susceptibility units. Published methods may report different parameterizations, including intrinsic compartment susceptibilities and signal fractions; these must be mapped to a common definition before numerical comparison. A small bulk value is compatible with several combinations of PCS and DCS and cannot be interpreted as proof of absent pathology (Figure 3). This cancellation concerns net susceptibility, not disappearance of all MRI signals: magnitude decay may remain sensitive to the underlying field of heterogeneity (9, 35).

**Figure 3:**
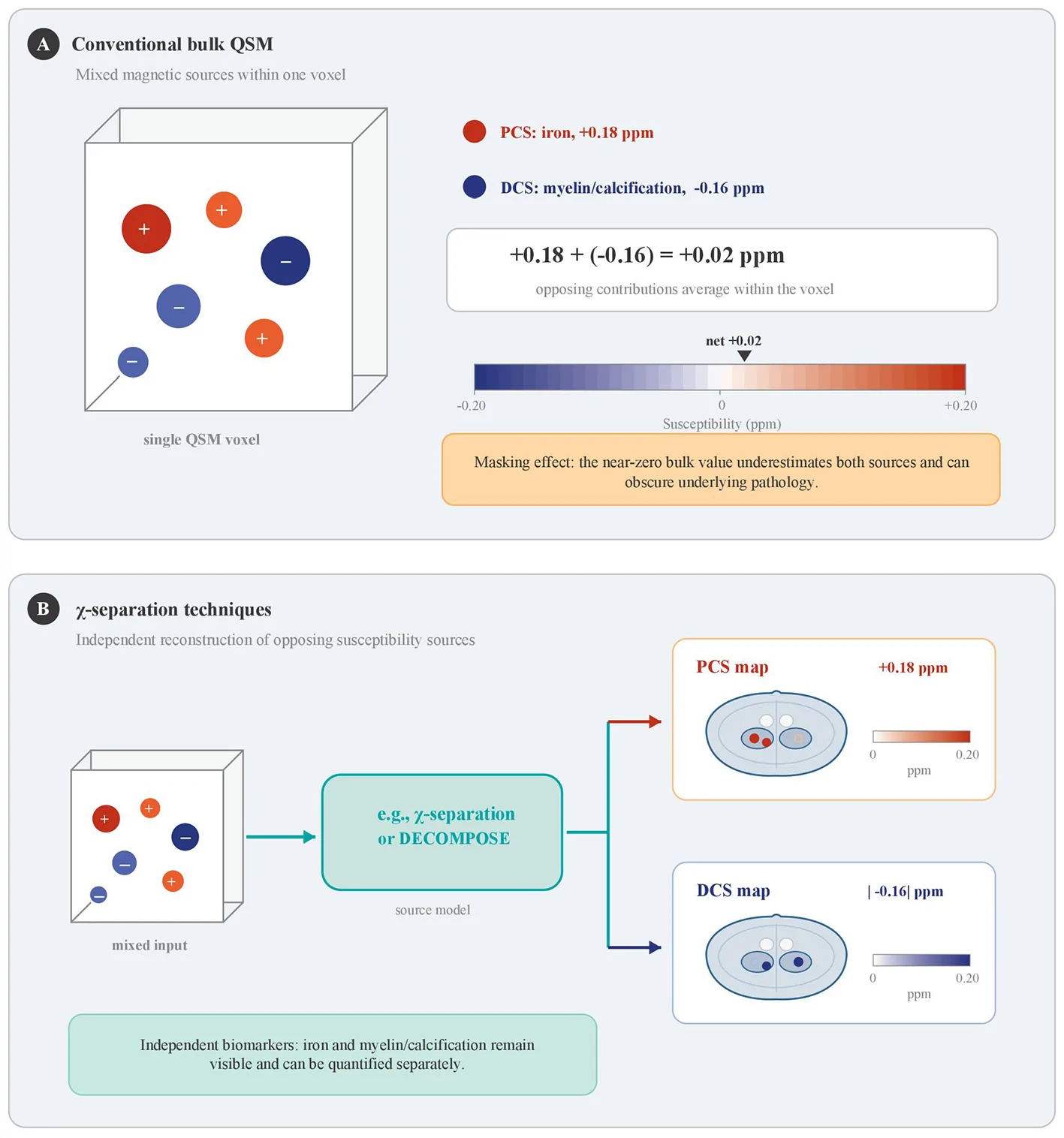
Opposing magnetic contributions can produce the same bulk susceptibility. **(A)** Three hypothetical voxel compositions share a bulk value of +0.02 ppm despite different PCS and DCS magnitudes. **(B)** Synthetic maps illustrate a mixed region with PCS = +0.18 ppm and DCS = −0.16 ppm, yielding bulk susceptibility of +0.02 ppm. PCS is shown on a nonnegative scale, DCS on a nonpositive scale, and bulk susceptibility on a diverging scale. These are prescribed illustrative contributions, not patient measurements or outputs from a fitted reconstruction. **(C)** Source separation needs information beyond the bulk value, together with a signal model and calibration. Component estimates do not independently identify the molecular sources or imply absence of reconstruction uncertainty.

### 2.3 Source-separation models and their assumptions

In the original χ-separation framework, local-field information is combined with reversible transverse relaxation, R2′ = R2^*^ – R2, to constrain paramagnetic and diamagnetic contributions (9). Separately acquired spin-echo data provide R2, while multi-echo GRE data provide field and R2^*^ estimates. The following equations describe an idealized two-source relaxation model, not a universal identity shared by every source-separation method. Positive constants of a_PCS_ and a_DCS_ relate the magnitude of each contribution to relaxation. Their values depend on calibration and the adopted microstructural model. For PCS and DCS expressed in ppm and relaxation rates in s^−1^, a_PCS_ and a_DCS_ have units s^−1^ ppm^−1^. A fitted R2^*^ model must also account for non-susceptibility baseline relaxation and other confounds; it must not substitute R2^*^ for R2′ without changing the model

$$\begin{array}{l} \chi_{\text{bulk}}\;=\;\text{PCS}\;+\;\text{DCS} \end{array}$$ (1)

$$\begin{array}{l} {\text{R}}_{2}^{\prime }\;=\;{\text{R}}_{2}^{*}\;-\;{\text{R}}_{2} \end{array}$$ (2)

$$\begin{array}{l} {\text{R}}_{2}^{\prime }\;\approx \;{\text{a}}_{\text{PCS}}\;\text{PCS}\;+\;{\text{a}}_{\text{DCS}}\;|\text{DCS}|. \end{array}$$ (3)

The additive susceptibility equation is a voxel-level conceptual representation. Reconstruction must still account for the nonlocal dipole field. Moreover, DECOMPOSE models a mixture of complex GRE signals, including compartment fractions, whereas APART-QSM uses iterative fitting and QSM-ARCS estimates relaxometric constants adaptively (35–37). Their outputs and assumptions should therefore be compared explicitly rather than treated as equivalent implementations of the same two linear equations.

PCS and DCS are magnetically defined, model-dependent measurements. Iron-bearing constituents can contribute to PCS, while myelin and some mineral or protein-containing structures can contribute to DCS. However, susceptibility also depends on chemical state, microstructural organization, and coexisting material. A lesion containing amyloid or tau is not necessarily uniformly diamagnetic. Neither a PCS increase nor a DCS decrease, by itself, identifies a unique cellular process or establishes the amount of a particular molecule (9, 38–40). To provide an illustrative example of the information contained in conventional QSM, R2^*^, and source-separated susceptibility maps, we performed a secondary analysis of publicly available MRI data from a non-neurodegenerative control participant in the OpenNeuro dataset ds007036 (41). Multi-echo GRE data were used to generate conventional QSM and R2 maps, followed by susceptibility source separation to estimate paramagnetic (χpara) and diamagnetic (χdia) components. Bilateral thalamic regions were delineated for visualization. This single-subject analysis was performed solely to illustrate the spatial relationship among the magnetic contrasts discussed in this review and was not used for statistical inference or to support disease-specific conclusions (Figure 4).

**Figure 4:**
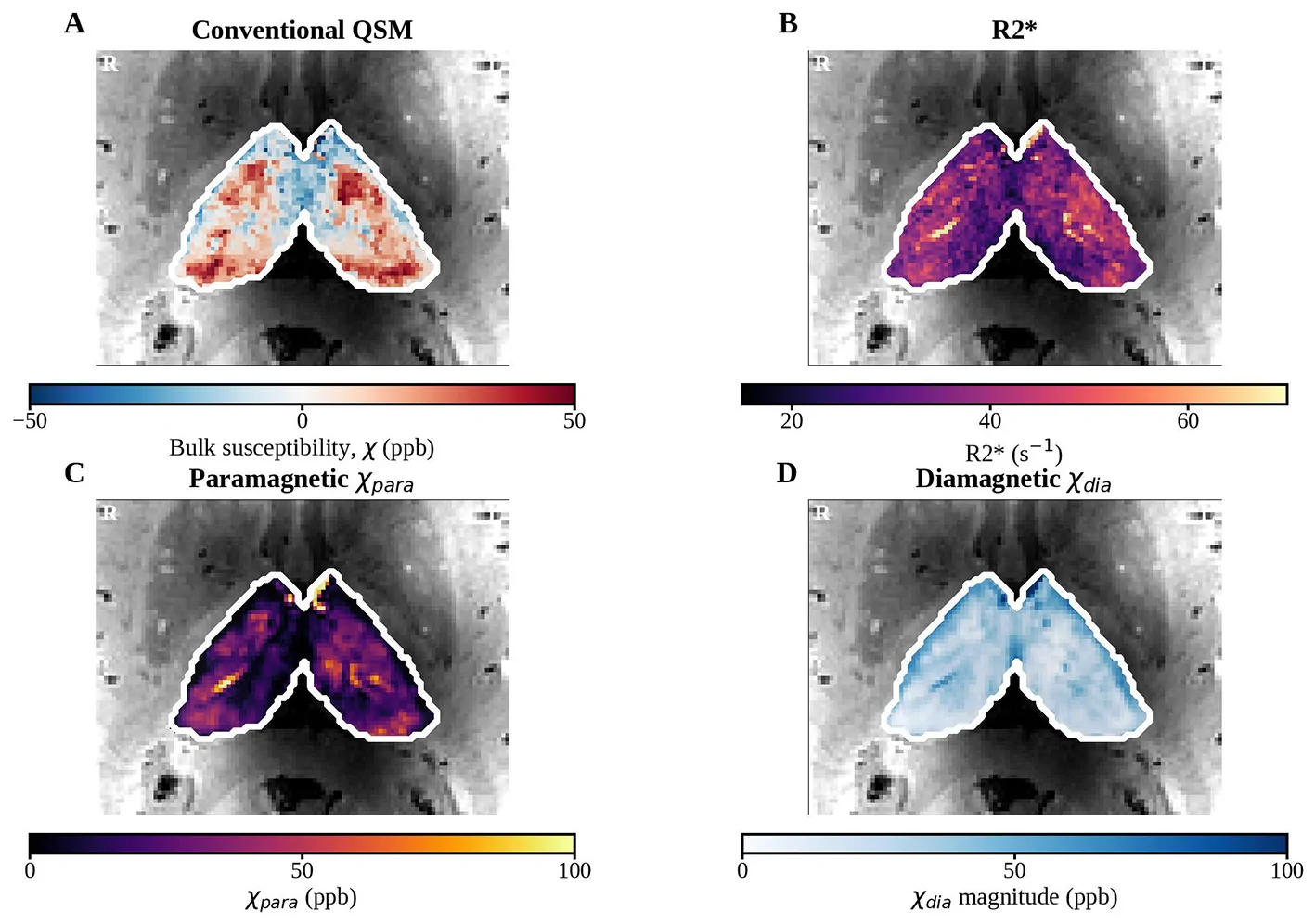
Thalamic susceptibility contrast across conventional QSM, R2* mapping, and χ-separation in a healthy control. The same axial slice through the bilateral thalamus is shown for one healthy participant (sub-H0101); the slice was selected objectively as the slice containing the largest bilateral thalamic cross-sectional area. The bilateral thalamic region is delineated by the white contour, with the corresponding quantitative map displayed within the ROI over the anatomical GRE T2*-weighted background. **(A)** Conventional quantitative susceptibility mapping (QSM), shown as whole-brain-mean-referenced bulk magnetic susceptibility, χ, in parts per billion (ppb) (100). Positive and negative values represent paramagnetic and diamagnetic bulk susceptibility, respectively. **(B)** R2* map derived from the nine-echo GRE acquisition using ARLO (101), expressed in s^−1^. **(C)** Paramagnetic component susceptibility (PCS; χpara) obtained using χ-separation with χ-sepnet-R2* (9, 43), displayed as a nonnegative susceptibility magnitude in ppb. **(D)** Diamagnetic component susceptibility magnitude (|DCS|; |χdia|) obtained from the same χ-separation reconstruction, displayed in ppb. Because the diamagnetic component is shown as its positive magnitude, larger values indicate a greater magnitude of the diamagnetic susceptibility contribution rather than a more positive bulk susceptibility. The comparison illustrates how conventional QSM reflects the net susceptibility arising from opposing paramagnetic and diamagnetic contributions, whereas χ-separation estimates their spatial contributions separately within the thalamus. R, right hemisphere. Data source: OpenNeuro dataset ds007036, subject sub-H0101, version 1.1.3 (doi: 10.18112/openneuro.ds007036.v1.1.3) (41).

GRE-only approaches reduce acquisition burden but replace separately measured relaxation information with additional modeling or learned relationships (35–37, 42, 43). In a direct MS comparison, R2^*^-based analytical separation showed offsets relative to R2′-based separation, while a learned method more closely matched its R2′-based counterpart (44). Such agreement supports technical feasibility within the studied setting; it does not demonstrate interchangeability across scanners or biological equivalence of the outputs. The normative atlas of 106 healthy participants provides reference distributions but does not supply disease-specific diagnostic thresholds (12).

Complementary measurements can constrain interpretation: myelin-sensitive magnetization transfer or myelin-water imaging for myelin hypotheses, computed tomography (CT) for mineralization, and appropriately selected positron emission tomography (PET) tracers or histology for protein-related hypotheses (38, 39, 45, 46). Each has its own resolution, specificity, and registration limitations. Correlation with one of these measurements strengthens a particular interpretation without converting PCS or DCS into a chemically selective assay.

## 3 Comparative evidence in thalamic applications

Table 1 summarizes the principal applications and the limits of their evidence. Table 2 identifies acquisition and analytical factors that should be compared before attributing conflicting findings to biology. These are qualitative assessments of the literature, not pooled effect estimates or formal certainty grades. Whole-thalamus averages, subnuclear measurements, and extra-thalamic findings are considered separately.

**Table 1:** Principal thalamic applications: observations and limits of inference.

Application/region	Observed evidence	Main limitation/justified inference
Development and aging/whole thalamus and subregions	Age-dependent, often nonlinear bulk-QSM patterns; large lifespan cohort (47–49, 53)	Age range and tissue sampling matter; cross-sectional trends do not identify an iron trajectory
MS/thalamus and pulvinar	Repeated bulk-QSM decreases; preliminary longitudinal source-separation evidence (57–60, 68)	Better-supported magnetic group difference; substrate and incremental prognostic value remain uncertain
AD/thalamus	Bulk-QSM differences inconsistent (71–73)	PET-associated source-separation findings are chiefly extra-thalamic (39); no validated thalamic protein assay
PD/thalamus	Often absent or heterogeneous bulk-QSM differences (76–80)	Nigral findings cannot establish thalamic PCS/DCS performance
ALS/thalamus	Susceptibility increases and severity associations in selected cohorts (4, 82–84)	Mostly observational evidence; association does not establish causal iron toxicity or monitoring utility
Calcification/thalamic lesions	Mixed deposits demonstrated in an experimental rat model (85)	Animal evidence; human PCS/DCS calcium specificity requires CT or histological validation
Niemann–Pick type C/pulvinar	Higher susceptibility in pulvinar clusters in 10 patients vs. 14 controls (86)	Single small 7 T cohort; regional, exploratory finding rather than a diagnostic biomarker

AD, Alzheimer's disease; ALS, amyotrophic lateral sclerosis; CT, computed tomography; DCS, diamagnetic component susceptibility; MS, multiple sclerosis; PCS, paramagnetic component susceptibility; PD, Parkinson's disease; PET, positron emission tomography; QSM, quantitative susceptibility mapping. Entries are a qualitative synthesis, not pooled effects or diagnostic recommendations.

**Table 2:** Dimensions to compare when interpreting heterogeneous thalamic QSM and source-separation findings.

Comparison dimension	How results can differ	Information needed for interpretation
Acquisition/field strength	Echo sampling, SNR, and local gradients alter estimates	B0, voxel size, TE range/spacing, motion handling; field-specific calibration
Reconstruction	Background removal and regularization change contrast and effect sizes	Software/version, mask, algorithms, parameters, sensitivity analyses
Susceptibility reference	A changing reference shifts relative target values	Reference definition and stability; comparison with a second plausible reference
Segmentation	Different atlases and registration include different tissues	Label definition, input contrast, registration QC, nucleus size vs. voxel size
Cohort characteristics	Age, phenotype, treatment, or vascular burden confound contrasts	Eligibility, age/sex distributions, disease stage, treatment and comorbidity
Atrophy/partial volume	CSF or white-matter contamination can alter regional means	Volume and susceptibility separately; boundary checks and partial-volume sensitivity
Design/validation	Cross-sectional, longitudinal, and experimental evidence answer different questions	Follow-up, multiplicity control, independent replication and tissue validation
Source-separation model	R2′-based and GRE-only outputs may differ in scale or offset	Signed convention, component/fraction definitions, relaxometric calibration and assumptions

B0, main magnetic field; CSF, cerebrospinal fluid; GRE, gradient-recalled echo; QC, quality control; SNR, signal-to-noise ratio; TE, echo time. These dimensions identify potential influences; they do not imply that every methodological difference reverses a study's result.

### 3.1 Development and aging

Thalamic susceptibility does not follow a single monotonic lifespan trajectory. Pediatric studies report increasing susceptibility with development, whereas adult studies describe nonlinear, absent, or negative associations depending on the age range and region examined (47–52). Treit et al. studied 498 healthy participants aged 5–90 years and demonstrated that QSM and R2^*^ provide different age-dependent information (53). These MRI trajectories should not be equated directly with trajectories of iron concentration: myelination, tissue water, reference choice, and microstructural organization also change with age.

Apparent disagreement between pediatric and older-adult cohorts is therefore not necessarily contradictory. A linear model fitted over a restricted interval may sample one portion of a nonlinear curve. Likewise, an age-related reference shift can alter relative thalamic susceptibility but should be assessed rather than asserted as an explanation for a particular study. Age and sex distribution, head orientation, and segmentation strategy should be compared before combining results (47, 48, 52–54).

Subnuclear analyses can reveal effects concealed by averaging; the pulvinar may differ from medial or lateral nuclei in baseline contrast and age associations (48). Conversely, a stronger apparent subnuclear effect may reflect smaller-sample variation or boundary contamination. Developmental source-separation observations, including a study of 65 healthy neonates, are consistent with spatially heterogeneous maturation, but do not establish diagnostic superiority for detecting delayed myelination or iron deficiency (55).

The most consistently supported conclusion is that age and anatomical sampling influence thalamic magnetic measurements. The chemical contribution to each trajectory, its longitudinal stability, and the predictive value of departures from a normative range remain uncertain. Cross-sectional age differences cannot alone establish within-person tissue loss or developmental rates.

### 3.2 Multiple sclerosis

Reduced whole-thalamus bulk susceptibility is repeatedly reported in MS, in contrast to the susceptibility increases often described in other deep gray-matter structures (56–63). Loss of a paramagnetic contribution could lower bulk susceptibility, whereas loss of a diamagnetic contribution could raise it; changes in both can coexist. Thus, a decrease is compatible with reduced iron-related susceptibility but does not independently prove iron depletion or demyelination. Distinct patterns in the pulvinar and other nuclei also argue against assuming uniform thalamic pathology (58, 63).

In the baseline MS-STAT2 imaging subgroup, Williams et al. compared 149 participants with secondary progressive MS and 33 controls at 3 T; lower thalamic susceptibility was associated with worse clinical measures and thalamic atrophy (59). Although recruited from a trial, this was a cross-sectional analysis and did not establish treatment response or prospective prognostic performance. Joint diffusion–QSM analysis also linked altered thalamic tissue properties with processing speed (64). A nonsignificant association with one lesion measure does not establish independence from all remote lesions or prove a specific microscopic substrate.

### 3.3 Source separation in MS: additional information and remaining ambiguity

Evidence from MS lesions supports sensitivity of source-separated measurements to differences in lesion state and longitudinal change (65–67). Much of this evidence concerns white-matter or cortical lesions rather than thalamic nuclei. It is relevant to biological interpretation, but thalamic remyelination monitoring cannot be regarded as validated on that basis. Differences in lesion age, inflammatory state, water content, and the chosen relaxation model may influence both components.

In a longitudinal study of 33 participants with relapsing-remitting MS and 34 controls, Xie et al. evaluated source-separated deep gray-matter measurements and susceptibility–volume products (68). Thalamic PCS mass was lower at follow-up, including a reduction relative to baseline. These supports considering regional volume alongside means susceptibility. However, multiplying a magnetic measurement by volume produces a susceptibility–volume index, not a direct mass of iron or myelin. It does not eliminate segmentation error, CSF partial volume, reference dependence, or assumptions about tissue composition. This single cohort therefore provides preliminary longitudinal thalamic evidence rather than definitive proof of chemically specific, atrophy-independent tissue depletion.

Heterogeneity should be investigated rather than explained by an untested narrative. Salman et al. could not reproduce the proposed early increase in thalamic susceptibility after closely matching a younger MS cohort and processing approach (60). Their subsequent methodological analysis examined 83 participants with MS and 44 controls across 154 reconstruction pipelines: thalamic susceptibility remained lower, while background-field removal, referencing, and segmentation influenced effect sizes more than dipole inversion in that dataset (69). These results weaken a universal “early-rise, late-decline” account and show that pipeline variability need not explain the direction of every discrepancy.

Taken together, decreased bulk thalamic susceptibility is more consistently supported than any single explanation for that decrease. PCS and DCS provide additional model-dependent information, but their incremental prognostic value over thalamic volume, conventional lesion measures, and bulk QSM remains to be established. Strong clinical claims require independent longitudinal cohorts with prespecified outcomes and acquisition-specific repeatability estimates.

### 3.4 Alzheimer's disease, protein-related hypotheses, and ARIA

Thalamic bulk-QSM findings in AD and mild cognitive impairment are inconsistent (70–73). Age distribution, diagnostic definition, disease stage, vascular comorbidity, reference choice, and the treatment of atrophy can change group comparisons. A cohort defined by amnestic symptoms is not equivalent to one with biomarker-confirmed AD, and vascular mild cognitive impairment should not be treated as a prodromal AD cohort (74). Evidence for a reproducible thalamic disease effect is weaker than the evidence for susceptibility alterations in several other deep gray-matter regions.

Ahmed et al. analyzed 49 amyloid- and tau-positive participants with amnestic dementia and 71 biomarker-negative cognitively unimpaired participants using 3 T GRE imaging, DECOMPOSE, and PET (39). Regional DCS findings and associations with tau PET support a relationship between magnetic measurements and AD-related tissue change. However, the reported PET correlation regions were primarily extra-thalamic; this study did not validate protein quantification within anterior, mediodorsal, or pulvinar nuclei. Associations with PET cannot distinguish a direct protein contribution from correlated myelin changes, iron redistribution, atrophy, or other pathology. The distinction is particularly relevant where MRI and PET have different spatial resolution and partial-volume effects.

Histological support for a protein-related interpretation is also model- and tissue-specific. Joshi et al. studied fixed brains from five PS19 tauopathy mice and five wild-type mice at 11.7 T, relating hippocampal and cortical susceptibility-component changes to immunohistology (40). This is small-sample, *ex vivo* animal evidence, not validation of human thalamic tau mapping. Neither this experiment nor the clinical AD study establishes DCS as an independent assay of amyloid or tau burden.

A role for χ-separation in amyloid-related imaging abnormality (ARIA) monitoring remains a hypothesis. Recommended MRI surveillance uses established sequences to assess edema/effusion and hemorrhagic changes, including FLAIR, T2^*^-weighted GRE or appropriate susceptibility-sensitive imaging, and diffusion imaging (75). The studies reviewed here do not establish incremental ARIA detection, grading, outcome prediction, or treatment-decision benefit from PCS/DCS, whether in the thalamus or elsewhere. Changes in edema, blood products, and tissue water would require explicit modeling and validation before source separation could be proposed as a monitoring endpoint.

### 3.5 Parkinson's disease and ALS

Thalamic bulk susceptibility in PD is often unchanged or inconsistent across cohorts, even when other structures show differences (76–80). Differences in motor subtype, cognition, disease stage, medication exposure, and regional sampling may contribute to heterogeneity; these explanations require direct testing. Evidence of source-separated changes in the substantia nigra, including prodromal cohorts, should not be presented as thalamic validation (81). Group discrimination in one structure does not establish a diagnostic threshold in another.

ALS studies have reported thalamic susceptibility increases and associations with clinical severity (4, 82–84). For example, Li et al. studied 34 patients and 34 controls and observed susceptibility alterations together with thalamic atrophy (4). This cross-sectional association is compatible with iron-related tissue change but cannot establish iron accumulation as the cause of disability or validate longitudinal monitoring. Evidence for thalamic PCS/DCS in ALS is less developed than bulk-QSM evidence; claims about separating microgliosis from tau in ALS would be extrapolations from a different disease model.

### 3.6 Calcification and other exploratory applications

Histologically verified iron- and calcium-positive deposits have been observed in rat thalamic lesions after experimental status epilepticus (85). This establishes a biological example of mixed mineral-related susceptibility sources in an animal model. It does not establish their frequency in human neurodegenerative thalamic disease, and coexistence within a nucleus is not itself proof of coexistence within the same imaging voxel. Mineral deposits in primary familial brain calcification can have complex composition (45, 46). PCS/DCS may help characterize magnetic heterogeneity, but identification and quantification of calcium require independent validation, commonly including CT or histology. Figure 5 summarizes the conceptual progression from conventional susceptibility-sensitive imaging to bulk QSM and source-separated component maps in the presence of mixed paramagnetic and diamagnetic contributions.

**Figure 5:**
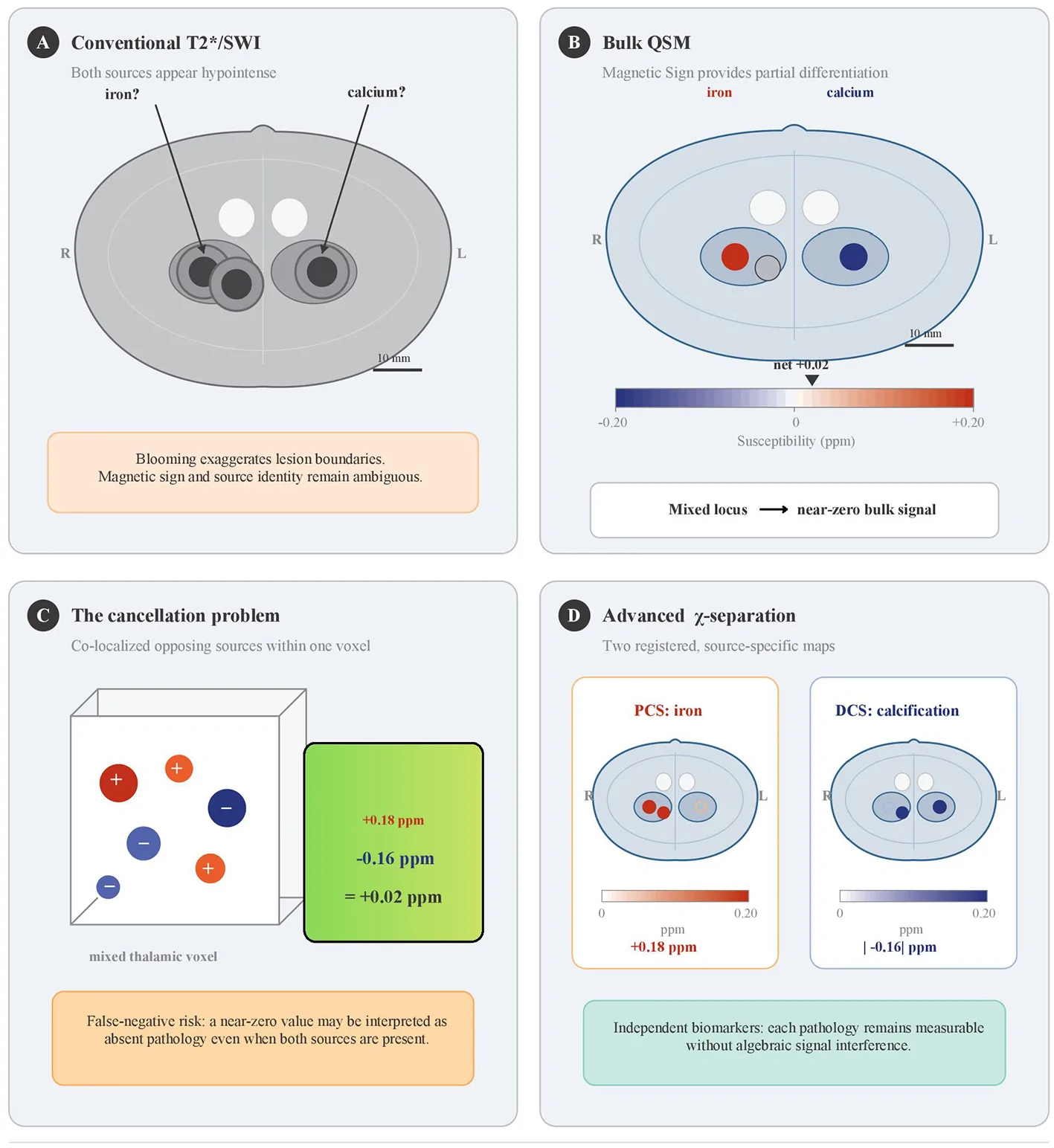
Conceptual progression from conventional susceptibility-sensitive MRI to bulk QSM and susceptibility source separation in the presence of mixed paramagnetic and diamagnetic sources. **(A)** On conventional T2*-weighted or susceptibility-weighted magnitude imaging, both iron-rich and calcified lesions may appear hypointense, while blooming can exaggerate apparent lesion extent; magnitude contrast alone does not establish source identity. **(B)** Bulk QSM adds susceptibility sign and can support magnetic differentiation when one source predominates. **(C)** When opposing susceptibility contributions coexist within the same voxel, partial cancellation can produce a deceptively small or near-zero bulk susceptibility value. **(D)** Susceptibility source-separation methods estimate separate paramagnetic and diamagnetic components, thereby reducing algebraic cancellation between opposing contributions. Iron and calcium are shown as illustrative source examples; PCS and DCS are magnetically defined measurements rather than chemically specific biomarkers, and tissue composition requires independent validation using appropriate complementary methods such as CT, PET, or histology.

The reviewed evidence does not validate χ-separation as a diagnostic test for primary familial brain calcification or as a measure of iron-mediated neurotoxicity. Other disease findings should likewise remain proportional to their support. A 7 T study in Niemann–Pick type C included 10 patients and 14 controls and reported higher susceptibility in pulvinar clusters associated with thalamic atrophy (86). This preliminary regional observation does not justify a general increase throughout the thalamus, nor classification of this lysosomal disorder as a neurodegeneration-with-brain-iron-accumulation syndrome.

### 3.7 Anatomical targeting: a limited adjacent application

Higher-resolution susceptibility imaging can aid visualization of deep nuclei (87–90). Nevertheless, QSM-assisted targeting and source-separation-based targeting are different claims. Ohtsuki et al. reported QSM and atlas-assisted ventral intermediate nucleus localization in a single patient with tremor and thalamic hypertrophy (91). This illustrates feasibility in an unusual case, not comparative surgical efficacy or validation of PCS/DCS-guided intervention. Reports on the subthalamic nucleus concern an anatomically separate target and should not be counted as evidence of thalamic biomarker performance (90, 92, 93).

## 4 Discussion: interpreting heterogeneous evidence

The principal contribution of susceptibility source separation is the estimation of additional magnetic information beyond a net QSM value. This may improve interpretation when opposing contributions coexist but increases reliance on relaxation modeling and calibration. The strongest thalamic disease evidence in the reviewed literature concerns group differences in MS; evidence for the chemical identity of those differences is less secure. AD protein mapping, calcification characterization, and treatment monitoring occupy different evidential levels and should not be presented as equally established applications.

Acquisition and analysis interact with the biology being studied (Table 2). Field strength changes frequency shifts, signal-to-noise ratio, and relaxation; intrinsic susceptibility itself should not simply be described as scaling with B0. Echo timing determines the observed decay regime, and voxel size controls partial volume. Reconstruction regularization can attenuate local contrast or spread information across nucleus boundaries. A reference shift changes all relative values, whereas segmentation may change the tissue included in only a particular region. These mechanisms are plausible contributors to heterogeneity, but should be demonstrated by sensitivity analyses rather than invoked after the fact to reconcile any disagreement (13, 44, 88, 94).

Cohort design is equally important. Age, sex, disease duration, phenotype, treatment exposure, vascular burden, and recruitment can differ between studies. Atrophy changes both anatomy and tissue sampling and cannot be managed adequately by labeling an association “atrophy-independent” without specifying the analysis. Mean susceptibility and volume should be reported separately, with susceptibility–volume products treated as complementary measures (68). Cross-sectional correlations indicate association, longitudinal data establish temporal change, and neither alone demonstrates causation. Multiple small-nucleus comparisons and reused cohorts also need to be considered when judging reproducibility.

### 4.1 Static dephasing and thalamic microstructure

The linear R2′ approximation is commonly motivated by the static dephasing regime (SDR), in which diffusion-driven averaging of microscopic field variations is negligible over the relevant signal evolution (9, 95). For a characteristic source dimension a, diffusivity D, and frequency shift Δω, a diffusion correlation time can be expressed as τD ∞ a^2^/D. The dimensionless quantity Δ*ωτ*D distinguishes limiting behavior: values much greater than one favor static dephasing, while values much less than one favor diffusion narrowing; intermediate values require attention to both mechanisms. These relations depend on geometry and should not be taken as universal diagnostic thresholds for a voxel.

The thalamus cannot be assumed to satisfy a pure SDR. Nuclei differ in source dimensions, fiber organization, cellular composition, and vascular contributions, while disease may change diffusivity and source distribution. Minimum echo time, echo spacing, and the fitted echo range affect which portion of nonexponential GRE decay is observed; spin-echo timing also affects diffusion-mediated dephasing. An acquisition protocol alone therefore does not prove that the thalamus is within the static limit.

Oliveira et al. acquired densely sampled GRE data at 3 T with a minimum echo time of 1.25 ms and reported nonexponential decay in subcortical gray matter, including the thalamus (94). Their modeling and simulations supported an intermediate regime with non-negligible diffusion effects. This finding cautions against applying a single relaxometric constant indiscriminately across nuclei or protocols. It does not show that every χ-separation estimate is invalid or provide a universal correction factor for diseased thalamic tissue.

Susceptibility anisotropy is a related but separate issue. Although the thalamus is gray matter, its intranuclear fibers and medullary laminae are not uniformly isotropic. Changes in head orientation may affect susceptibility, relaxation, and the contribution of adjacent white matter. The magnitude of these effects has not been established uniformly across thalamic nuclei. Neither strong anisotropy everywhere nor its complete absence should be assumed (9, 96).

Susceptibility tensor imaging can model orientation-dependent susceptibility, but normally requires multiple head orientations; approaches such as DeepSTI seek to reduce this burden (97–99). These methods are relevant technical developments, rather than established solutions for routine thalamic disease assessment. Longitudinal studies should document head positioning and test whether observed changes exceed measurement variability.

Higher B0 does not by itself invalidate the SDR. With source size and diffusivity fixed, an increased frequency shift can move a system toward the static limit. Separate difficulties at 7 T include shortened T2^*^, loss of later-echo signal, field inhomogeneity, phase wrapping, and transfer of relaxometric constants or learned mappings calibrated at 3 T (88, 89, 94). These effects must be distinguished from the physical criterion defining SDR.

### 4.2 From research measurements to clinical validation

Biological sensitivity means that a measurement changes with some aspect of tissue state. Improved interpretability means that additional measurements constrain competing explanations. Potential clinical utility requires evidence that the information could improve a specified diagnostic, prognostic, or monitoring decision. A clinically validated biomarker additionally requires reliable measurement, validation for that intended use, and evidence that relevant thresholds or changes generalize to the target population. For thalamic PCS/DCS, standardized protocols, pathological specificity, clinically useful thresholds, and independent multicenter validation remain insufficiently established. Prospective studies should compare source separation against bulk QSM, structural volume, and relevant clinical or multimodal benchmarks, rather than assume benefit from the availability of two maps.

## 5 Conclusions

Thalamic QSM is sensitive to magnetic tissue differences, but a regional susceptibility change has several possible biological and methodological explanations. Source separation can estimate opposing magnetic contributions that are obscured in a net value, subject to acquisition and model assumptions. PCS and DCS must remain distinct from claims about specific molecules or pathological processes. Current evidence supports their use as research measurements for testing tissue hypotheses, with the most developed thalamic disease evidence in MS. Protein-related mapping, mixed-mineral characterization, and treatment monitoring require further direct validation. Clinical adoption should depend on reproducible added value for a defined use, rather than on presumed chemical specificity or the visual detail of the maps.
